# Supplementary material for: The causal relationship between the human gut microbiota and pyogenic arthritis: a Mendelian randomization study
Source: Front Cell Infect Microbiol. 2024 Nov 26;14:1452480. doi: 10.3389/fcimb.2024.1452480 (PMC11629706; doi:10.3389/fcimb.2024.1452480)
Supplement: Supplementary file 1 [file DataSheet1.zip › mendelian test/ebi-a-GCST90027511.csv_leave_one_out.pdf]

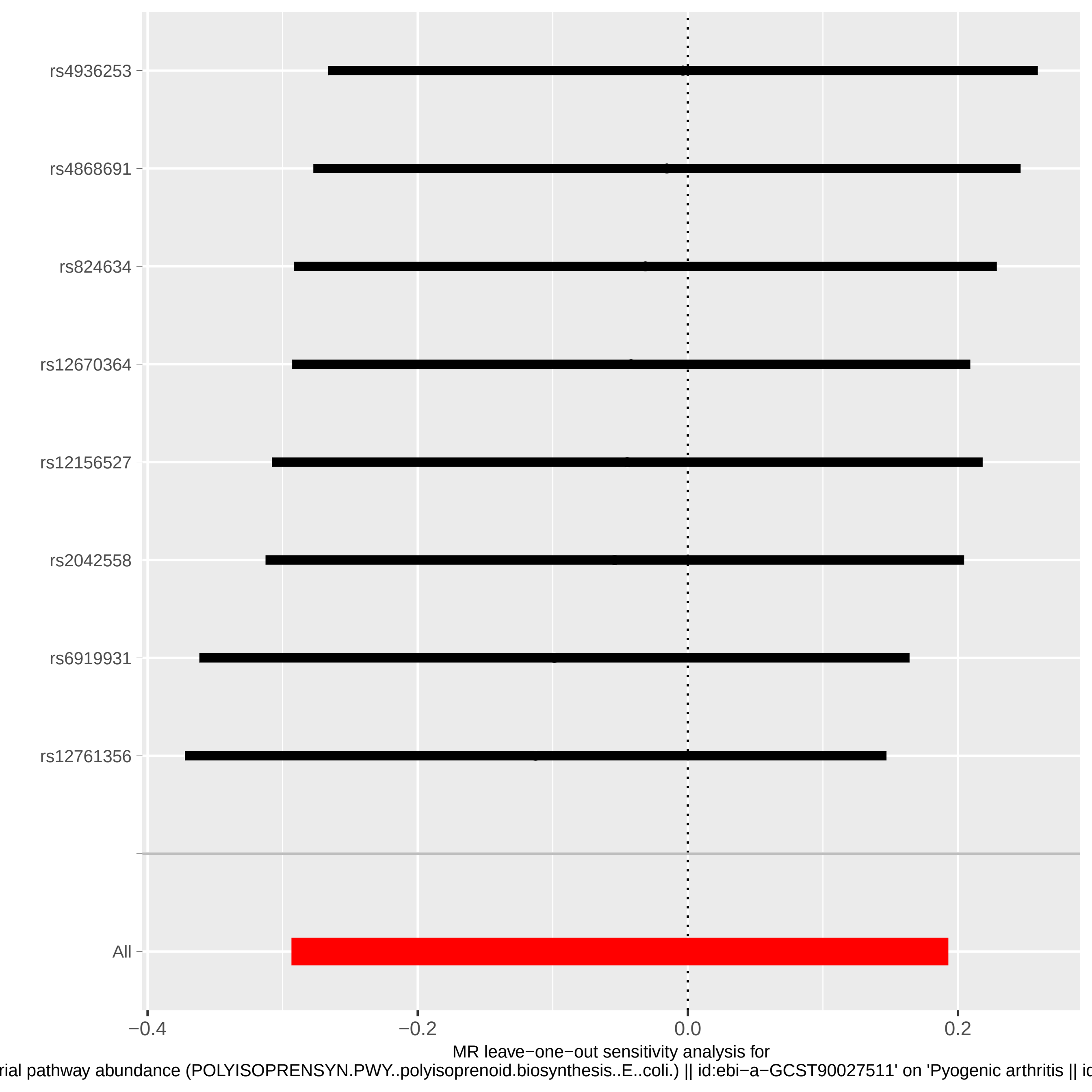
rs4936253
rs4868691
rs824634
rs12670364
rs12156527
rs2042558
rs6919931
rs12761356
All
−0.4
−0.2
0.0
0.2
MR leave−one−out sensitivity analysis for
rial pathway abundance (POLYISOPRENSYN.PWY..polyisoprenoid.biosynthesis..E..coli.) || id:ebi−a−GCST90027511' on 'Pyogenic arthritis || id
